# Magnesium—A More Important Role in CKD–MBD than We Thought

**DOI:** 10.3390/diagnostics12040880

**Published:** 2022-04-01

**Authors:** Ileana Peride, Mirela Tiglis, Tiberiu Paul Neagu, Andrei Niculae, Ionel Alexandru Checherita

**Affiliations:** 1 Clinical Department No. 3, “Carol Davila” University of Medicine and Pharmacy, 050474 Bucharest, Romania; al.checherita@gmail.com; 2 Clinical Department No. 14, “Carol Davila” University of Medicine and Pharmacy, 050474 Bucharest, Romania; mirelatiglis@gmail.com; 3 Clinical Department No. 11, “Carol Davila” University of Medicine and Pharmacy, 050474 Bucharest, Romania; dr.neagupaul@gmail.com

**Keywords:** chronic kidney disease, serum magnesium level, risk factors, progression, outcome

## Abstract

Chronic kidney disease (CKD) is associated with different complications, including chronic kidney disease–mineral and bone disorder (CKD–MBD), which represents a systemic disorder that involves the presence of different mineral or bone structure abnormalities (i.e., modification of bone turnover, strength, volume, etc.), including even vascular calcification development. Even if, over the years, different pathophysiological theories have been developed to explain the onset and progression of CKD–MBD, the influence and importance of serum magnesium level on the evolution of CKD have only recently been highlighted. So far, data are inconclusive and conflicting; therefore, further studies are necessary to validate these findings, which could be useful in developing a better, more adequate, and personalized management of CKD patients.

## 1. Introduction

Chronic kidney disease (CKD) is defined as kidney structure or function abnormalities (i.e., albuminuria, abnormalities in the urine sediment, tubular disorders, histological/imaging anomalies, etc.) present for more than 3 months. An important aspect in the evaluation of the renal impairment degree is the use of estimated glomerular filtration rate (eGFR) based on different formulas, usually using serum creatinine. The threshold at which eGFR is considered decreased is 60 mL/min/1.73 m^2^, and kidney failure is defined as GFR <15 mL/min/1.73 m^2^ [1].

In addition, CKD, with a global prevalence of almost 11–13%, is considered an independent risk factor for cardiovascular disease (CVD), with increased risks of cardiovascular morbidity and premature mortality, leading to a reduced quality of life [2].

In addition to the aforementioned increased cardiovascular risk, CKD is also associated with other complications, such as anemia [3], dyslipidemia [4], and chronic kidney disease–mineral and bone disorder (CKD-MBD) [5].

Even though an association between kidney disorders and bone abnormalities dates back to 1883, when, in patients with albuminuria and bone malformations, Lucas proposed the term “renal rickets” [6], the currently accepted CKD–MBD definition, from pathogenesis to identification, dates back to 2006 as a systemic disorder that involves the presence of different mineral/vitamin (i.e., calcium, phosphorus, vitamin D, etc.) or bone structure abnormalities (i.e., modification of bone turnover, strength, mineralization, etc.), including even vascular calcification development [7].

## 2. Methods

For the present review, we consulted the PubMed and Google Scholar databases, introducing different terms for general characterization, such as “serum magnesium”, “serum magnesium and chronic kidney disease”, “hypomagnesemia”, “hypermagnesemia”, “serum magnesium and renal transplant”, and “magnesium levels in chronic kidney disease patients”. In addition, in order to present better and updated information related to the recent knowledge regarding magnesium influence on chronic kidney disease patients, we added supplementary filters, such as “publication date in the last 5 years”.

## 3. The Correlation between Serum Magnesium and Chronic Kidney Disease

Traditionally, it was considered that the first detectable mineral metabolism abnormality in CKD patients that might explain the onset of hyperparathyroidism was the increase in serum phosphate, which determined hypocalcemia, the decrease in calcitriol, and consequently the augmentation of PTH values [8]. It was found that a high level of PTH is the body’s response to maintain normal serum calcium and phosphate amounts, by increasing urinary phosphorus excretion. Once renal impairment progresses, serum phosphorus values increase further (an increase that cannot be urinary excreted due to the extensive irreversible lesions in the kidney), and the excess binds the bioavailable calcium, resulting in calcium hydrogen phosphate (CaHPO_4_) that leads, in this manner, to the onset of hypocalcemia. This hypocalcemic state contributes to a further elevation of serum PTH values that determines an inadequate regulation of serum phosphate, calcium, and calcium phosphate product [9,10].

**Figure 1:**
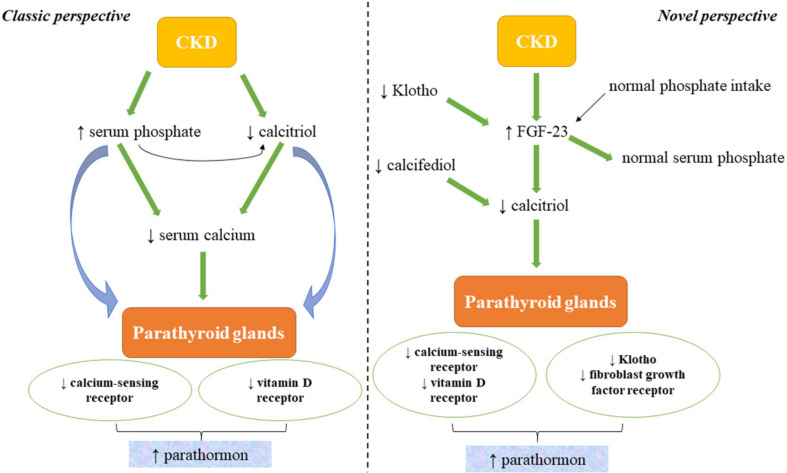
The classic and novel perspectives of CKD–MBD pathophysiology (modified after [8]). CKD: chronic kidney disease; FGF-23: fibroblast growth factor 23; calcifediol: 25-hydroxyvitamin D; calcitriol: 1,25-dihydroxyvitamin D.

The overall result is that PTH increases as renal function decreases; therefore, secondary hyperparathyroidism (SHPT), theoretically, should only be present in later stages of CKD, which is not the case in clinical practice. For this reason, in the last decades, intensive experimental and clinical trials were conducted in order to determine whether other triggers could be incriminated. Over the years, different pathophysiological theories have been presented; however, once fibroblast growth factor 23 (FGF-23) and Klotho were discovered, it could be explained why, in the absence of hyperphosphatemia (normal values of phosphate being maintained by the increased levels of FGF-23 in the initial stages of CKD), PTH levels would increase (Figure 1) [8,11].

**Figure 2:**
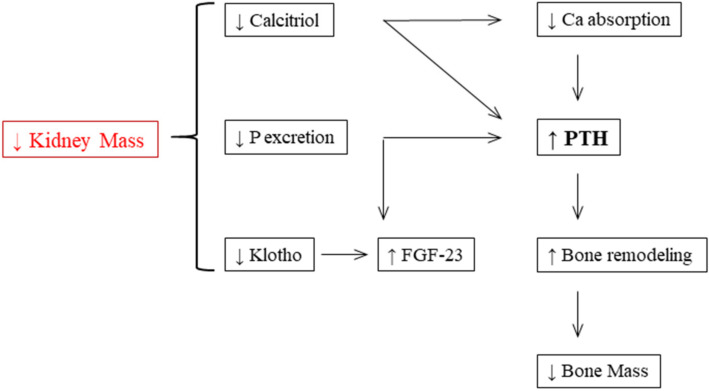
The pathophysiology of CKD-MBD (modified after [11]). P: phosphate; FGF-23: fibroblast growth factor 23; Ca: calcium; PTH: parathyroid hormone.

Maintaining phosphate homeostasis is the main function of the endocrine hormone FGF-23. In the bone, FGF-23 is predominantly synthesized by osteocytes. Calcitriol, phosphate, and PTH represent the main stimulators of FGF-23 secretion. Additionally, calcitriol directly activates the expression of FGF-23 by binding to the vitamin D receptor and subsequent stimulation of the promoter region of FGF-23, while it is unknown which mechanisms affect the expression of FGF-23 by phosphate. In the kidney, FGF-23’s physiological functions are regulated by binding to a fibroblast growth factor receptor (FGFR) complex and its unique Klotho coreceptor. By activating the FGFR1–Klotho complex, FGF-23 decreases renal phosphate reabsorption [12]. In summary, the expression of Klotho in the kidney decreases and that of FGF-23 increases as CKD advances. As calcitriol decreases and PTH subsequently rises, higher FGF-23 levels sustain serum phosphate at normal levels until the final stages of CKD (Figure 2) [11,13].

In addition to the well-known and studied factors that contribute to the onset and progression of CKD–MBD, a new “player”, serum magnesium (Mg), attracted the attention of the scientific community in better understanding the complex pathophysiological mechanisms underlying this condition, and finding better and more suitable forms of treatment, as the use of non-calcium-based phosphate binders and calcimimetics is challenging in daily practice [14,15]. The available data, albeit not unified, showed in CKD patients that both hypomagnesemia and hypermagnesemia can induce a deleterious effect on mineral and bone disorders, associated with increased risk of mortality [14]. Therefore, a recent study identified the fraction excretion (FE) rate of different electrolytes, including Mg, in 762 patients diagnosed with different CKD stages. A significant statistically difference was noticed among all CKD stages regarding FE Mg mean values, results that could represent a valuable contribution to a better understanding of the magnesium influence on CKD patients [16].

- CKD stage 1—FE Mg (%) = 2.24 ± 1.45,
- CKD stage 2—FE Mg (%) = 3.30 ± 3.01,
- CKD stage 3a—FE Mg (%) = 4.67 ± 4.61,
- CKD stage 3b—FE Mg (%) = 7.52 ± 5.23,
- CKD stage 4—FE Mg (%) = 11.70 ± 8.16,
- CKD stage 5 (including dialysis patients)—FE Mg (%) = 22.43 ± 12.79.

These data (increased FE Mg levels especially in CKD stage 5) also showed the possibility that the increase of filtered magnesium presents a higher impact on FE Mg, regardless of the PTH effect, contributing in this manner to an early diagnosis of renal impairment. In addition, serum Mg mean level was slightly increased in CKD stage 5 patients [16].

- CKD stage 1—Mg (mg/dL) = 2.03 ± 0.24,
- CKD stage 2—Mg (mg/dL) = 2.03 ± 0.29,
- CKD stage 3a—Mg (mg/dL) = 1.99 ± 0.45,
- CKD stage 3b—Mg (mg/dL) = 2.19 ± 0.54,
- CKD stage 4—Mg (mg/dL) = 2.21 ± 0.48,
- CKD stage 5 (including dialysis patients)—Mg (mg/dL) = 2.40 ± 0.59.

Another recent study, performed on non-dialysis CKD patients, showed that FE Mg levels are correlated with PTH values (increased FE Mg values were associated with elevated PTH levels), probably because, considering that FE Mg levels reflect the state of tubular function, the included subjects did not have associated tubular dysfunction. In summary, in patients with preserved tubular function, CKD progression is linked to increased PTH and decreased calcium levels, but normal levels of Mg, due to the compensatory mechanism induced by FE Mg [17].

Regarding renal transplant patients, hypomagnesemia can be noticed several years after transplantation, but especially in the first weeks. In addition, a study conducted on 320 subjects concluded that a low serum magnesium level (0.68–0.78 mmol/L) could be associated with a higher risk of allograft dysfunction, even loss. It appears that the presence of hypomagnesemia in this group of patients could be induced by specific immunosuppressive medication [18].

## 4. The Importance of Magnesium in the Body

Magnesium (Mg), an important cation of the body [19,20], despite being involved in more than 300 enzymatic reactions [21] and playing an important role in mineral bone and lipid metabolism, neurotransmitter release, oxidative stress prevention, and regulation of heart rhythm and vascular tone [22,23,24], has not received the needed attention in clinical practice compared to calcium, phosphorus, and potassium have.

Magnesium is an alkaline element that has the following characteristics [21]:

- An atomic weight of 24,305 Da,
- A melting point of 648.8 °C,
- A boiling point of 1090 °C,
- A hexagonal crystal structure,
- Three stable natural isotopes,
- The property to strongly bind water in its dissolved state.

Therefore, once hydrated, it is difficult to be dehydrated, and this feature can explain the reason why Mg, despite presenting similar chemical properties to calcium, cannot easily pass the narrow channels of the membranes [21].

Of the total body magnesium (almost 20 mmol/kg of fat-free tissue) [18], 99% is divided across bone (60–65%), muscle (25–30%), and other soft tissues, with an average amount of 25 g in a healthy adult individual [21,25]. It is important to highlight that, even if with ageing there is a decrease in the magnesium bone content, one-third of this skeletal Mg can be exchangeable, maintaining in this manner an adequate value of the serum Mg [21]. In the intravascular compartment, less than 1% of the total body Mg is present, and serum concentrations vary from 0.65 to 1.05 mmol/L (1.4–2.0 mEq/L or 1.7–2.4 mg/dL), divided as ionized (55–70%), protein-bound (20–30%), and anion-complexed (5–15%) forms such as bicarbonate, sulfate, citrate, or phosphate [21,26,27]. In order to maintain a suitable magnesium balance in normal adults, daily magnesium intake should be between 0.3 and 0.35 mEq/kg [28].

Approximately 30–40% of the ingested Mg is intestinally absorbed, through paracellular and transcellular pathways. With dietary deficits, fractional magnesium absorption can increase as high to as much as 80% and decrease to as little as 20% in the case of magnesium excess. The passive, paracellular pathway is responsible for 90% of intestinal Mg absorption, with the remaining 10% being absorbed through transient receptor potential melastatin 6 and 7 (TRPM6 and TRPM7), an active mechanism [29].

**Figure 3:**
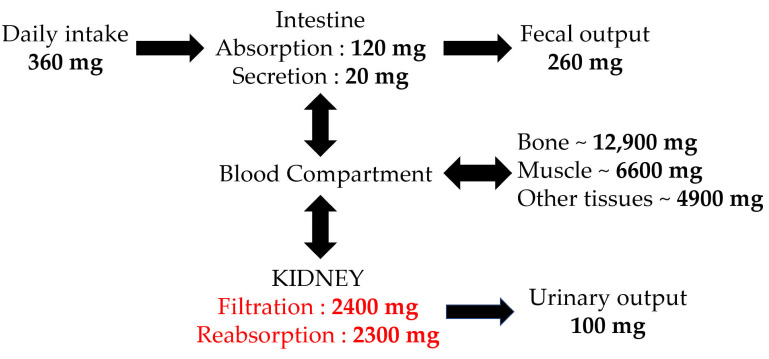
Magnesium homeostasis ([27]).

**Figure 4:**
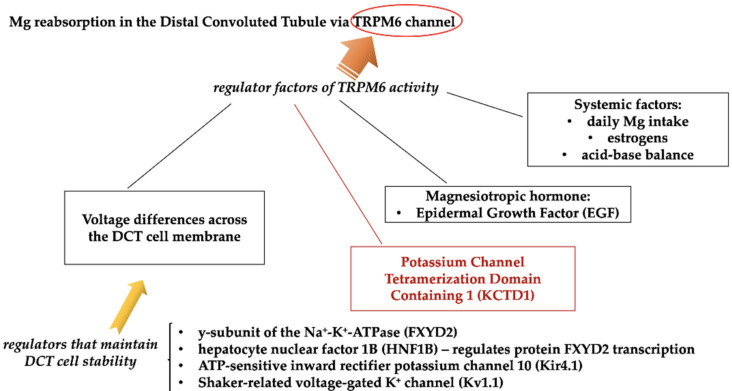
Magnesium regulatory system within the distal convoluted tubule (modified after: [33]). TRPM6: melastatin-related transient receptor potential cation channel 6.

The key magnesium excretory pathway is the kidney, where fractional excretion varies from almost 0.5% in hypomagnesemia to more than 70% in the case of hypermagnesemia. Most of the filtered magnesium is reabsorbed in the loop of Henle [30]; with passive paracellular magnesium uptake, bulk magnesium transport (up to 70%) occurs in the thick ascending limb with the help of a luminal positive gradient driven by potassium recycling via the apical NKCC2 (Na^+^–K^+^–2Cl^−^ cotransporter) and ROMK (renal outer medullary potassium channel) transporters [31]. Magnesium reabsorption fine-tuning occurs in the distal convoluted tubule (DCT), representing the final site of serum Mg reabsorption, and it is a TRPM6 (melastatin-related transient receptor potential cation channel 6)-driven active process, partly under the control of PTH (which increases the cation’s tubular reabsorption), epidermal growth factor, and estrogen (both activating TRPM6 transcription) [32]. Recently, a new factor was identified as the major regulator of the TRPM6 activity—KCTD1 (potassium channel tetramerization domain containing 1), representing a BTB domain-containing nuclear protein (BTB = Broad-Complex, Tramtrack, and Bric-à-brac) [33,34]. TRPM6 expression and activity seem to also be altered by alkalosis, acidosis, cyclosporine, and thiazide diuretic [35]. A summary of magnesium homeostasis is shown in Figure 3 [27], and magnesium transporters and regulators within DCT are presented in Figure 4 [33].

As already described in Figure 3, an important amount of serum Mg is provided by the skeletal system (almost 50% of the bodily magnesium being stored at this level). It appears that the transporters and regulators involved in the storage and exchange of Mg within the bone are similar to those for calcium, but the exact regulatory system has not been identified (magnesium mobilization from the bone probably also depends on the transient receptor potential channel family, as well as the agonist effect for the calcium-sensing receptor) [33,36].

**Figure 5:**
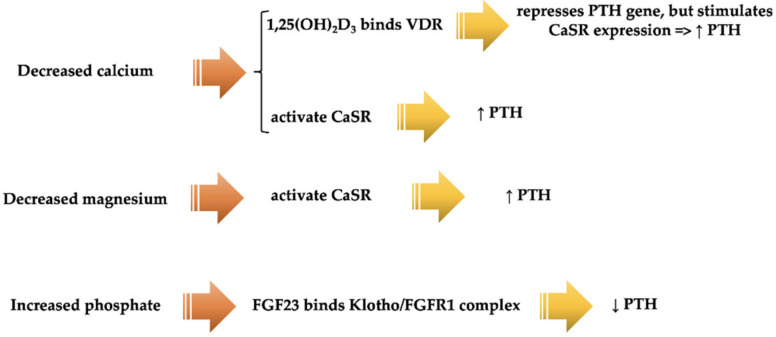
PTH regulatory system within the parathyroid gland, in the case of decreased calcium and magnesium, and increased phosphate (modified after [36]). 1,25(OH)_2_D_3_: vitamin D3 1,25-dihydroxyvitamin D; VDR: vitamin D receptor; PTH: parathyroid hormone; CaSR: calcium-sensing receptor; FGF23: fibroblast growth factor 23; FGFR1: fibroblast growth factor receptor 1.

It is important to emphasize that Mg represents a type I agonist for CaSR (calcium-sensing receptor), as previously mentioned; it can activate CaSR independently of the extracellular calcium absence. Therefore, CaSR can also control magnesium homeostasis within the body, and PTH can be influenced by the levels of serum Mg. Figure 5 describes the PTH regulatory system within the parathyroid gland, highlighting also the influence of serum magnesium in PTH expression [36].

Although magnesium has various and important roles in the organism, being implicated in more than 300 enzymatic ATP-dependent (adenosine triphosphate-dependent) reactions [21], the assessment of magnesium is often overlooked in daily practice. In addition, it should be acknowledged that magnesium presents a significant contribution in maintaining the normal homeostasis of the body. As already mentioned, magnesium participates in ATP metabolism, vascular tone and heart rhythm regulation [37], contraction and relaxation of the muscles (as a calcium antagonist), maintaining a normal neurological function, insulin secretion and cellular glucose metabolism [38,39], and bone formation [21]. Therefore, it is important to remember the possibilities to monitor the levels of Mg: (1) assessing the Mg content in serum (the most used and inexpensive method), erythrocytes, leucocytes, or muscle, with the latter representing an expensive method; (2) metabolic assessment (i.e., renal excretion, balance or isotopic analysis, Mg retention test); (3) the evaluation of free Mg (i.e., spectroscopy using nuclear magnetic resonance, ion-selective electrodes, and fluorescent dyes for magnesium that are rarely used due to a higher affinity of the fluorescent probes for calcium) [21]. Regarding the renal excretion method to determine the absorption and excretion of Mg, it should be emphasized that, for an optimal and reliable evaluation, a 24 h urine probe is required to be collected. A low urine excretion indicates decreased diet intake or absorption of this element; in contrast, a high urine excretion shows Mg renal wasting [21].

Considering the importance of magnesium in the body, it is essential to recognize the signs and symptoms induced by the decrease (hypomagnesemia) or increase (hypermagnesemia) in serum Mg values.

### 4.1. Hypomagnesemia

Usually, hypomagnesemia is considered for a serum Mg level below 0.61–0.75 mmol/L [15]. The recognized causes for the presence of hypomagnesemia are as follows [21]:

- Metabolic disorders—diabetes mellitus [40],
- Malnutrition,
- Gastrointestinal dysfunctions (malabsorption or loss—neoplasia, cirrhosis, severe diarrhea, etc.),
- Renal impairment (tubulointerstitial nephropathies—congenital or acquired),
- Genetic disorders—familial hypomagnesemia associating nephrocalcinosis due to CLDN16 gene mutation [41],
- Endocrine diseases—hyperaldosteronism (primary and secondary), syndrome of inappropriate antidiuretic hypersecretion, functional hypoparathyroidism [42], hungry bone disease (post parathyroidectomy),
- Cerebrovascular diseases,
- Drug-related—antibiotics (i.e., aminoglycosides), immunosuppressive therapy (i.e., cyclosporine, tacrolimus), chemotherapy (i.e., cisplatin, cetuximab, 5-fluorouracil) [43], antifungal medication (i.e., amphotericin B), proton pump inhibitors (i.e., omeprazole) [44,45], antiviral therapy (i.e., foscarnet), and diuretics. Diuretics, especially loop diuretics (i.e., furosemide, torasemide, bumethanide, etc.), which are responsible for increased urine excretion, contribute the most in the onset of hypomagnesemia, but long-term administration of thiazide diuretics can also have the same effect. Osmotic agents (i.e., mannitol) and potassium-sparing diuretics (i.e., triamterene, amiloride) increase the level of serum magnesium,
- Miscellaneous—severe burns, alcoholism, stress, cardiopulmonary bypass surgery, etc.

Furthermore, a severe decreased amount of serum magnesium, according to various studies, can contribute to an elevated risk of mortality and morbidity; therefore, it is essential to assess the level of serum Mg in our patients, especially in critical ill patients [46,47] associated with a vast range of comorbidities (cardiovascular, neurological, renal, metabolic dysfunction, neoplasia, infection with SARS-CoV-2, etc.), as well as in other types of patients, such as chronic dialyzed or diabetic individuals [21,48,49]. Considering the involvement of magnesium in the control of glycemia, as previously mentioned, a recent study of Zahra et al., involving 101 diabetic patients, showed the association between hypomagnesemia and a poor glycemic control, as well as long-term complications related to diabetes mellitus (especially with the development of diabetic nephropathy) [50]. A study performed by Shivakumar et al. also revealed a correlation between hypomagnesemia and another long-term diabetic-related complication, diabetic retinopathy [39].

Due to the COVID-19 pandemic, often associated with severe respiratory infection and, consequently, systemic inflammation, different studies proposed the administration of protective agents against infection that could allow a better control of the inflammatory syndrome, which could increase the survival rate [51,52]. Considering that vitamin D can protect the organism against respiratory infections [53], magnesium can stimulate vitamin D’s beneficial effects [54], in addition to its bronchodilator role, and vitamin B12 modulates normal gut microbiota, a recent study focused on the roles of these elements and noticed the beneficial effects of supplementation with vitamin D, cobalamin (vitamin B12), and magnesium in COVID-19 individuals; patients who did not receive these supplements were more likely to require oxygen therapy [51]. Therefore, the present recommendations for the management of COVID-19 patients also include the evaluation of serum magnesium level, with supplementation upon identifying a decrease in serum value [55].

In addition, several studies have indicated a clear association between hypomagnesemia and cardiovascular mortality risk [56], as well as the development of different cardiovascular diseases [48], such as postoperative atrial fibrillation in patients who underwent surgery for esophageal cancer [57], diastolic cardiomyopathy [58,59], and acquired long QT syndrome [60].

The frequent clinical symptoms and signs, along with laboratory findings, noticed in the case of hypomagnesemia manifest as follows: neuromuscular (muscle fasciculation, weakness, positive Chvostek and Trousseau signs, and dysphagia), cardiac (arrhythmias), neurological (depression, nystagmus, agitation, and seizures), hypokalemia, and hypocalcemia [21].

In order to prevent these possible manifestations, as well as prevent the development or worsening of different pathologies (mentioned above), the treatment consists of oral magnesium supplementation for mild cases, and intravenous magnesium administration for patients with severe hypomagnesemia to rapidly correct this deficiency [21]. In addition, considering the association among cardiovascular diseases, diabetic mellitus, and gastrointestinal disorders, recent findings suggest a magnesium rich-diet (i.e., spinach, whole grains, beans, nuts, avocado, seafood, etc.) or, when due to different possible associated conditions (i.e., chronic kidney disease) or insufficient Mg diet intake, 600 mg/day magnesium-based supplements [61]. Furthermore, an assessment of serum magnesium level every 6 months is indicated, especially in patients with known cardiovascular, gastrointestinal, renal, or metabolic disorders [61].

### 4.2. Hypermagnesemia

Most cases of hypermagnesemia (a serum magnesium level above 2.5 mg/dL [62]) are noticed in elderly and/or chronic kidney disease patients [21]. Possible causes of the serum magnesium augmentation could be important oral administration of magnesium-containing drugs or salts (i.e., laxatives, antacids), infusion with magnesium sulfate, or high doses of magnesium supplementation, along with an suboptimal assessment of serum Mg levels.

The clinical milieu of hypermagnesemia includes different types of manifestations: neurological (i.e., muscle weakness, loss of deep tendon reflexes, paralysis, lethargy, confusion, and coma), cardiovascular (i.e., from mild to profound hypotension, cardiac arrest, bradycardia, tachycardia, and arrythmias), respiratory (i.e., increased respiratory rate, apnea, and respiratory arrest), and gastrointestinal (i.e., paralytic ileus and nausea).

Depending, on the intensity of hypermagnesemia, the treatment requires cessation of magnesium supplementation or administration, the use of calcium gluconate, and even the initiation of renal replacement therapy in severe cases. It is important to correctly define hypermagnesemia, as, similar to hypomagnesemia, this condition can also be associated with a high risk of mortality, especially in critical ill patients presenting different severe pathologies (i.e., renal impairment and neoplasia) [63,64].

## 5. Magnesium Disorders in CKD

When it comes to Mg and CKD, many questions remain unanswered. Despite the renal function deterioration being recognized as a regular prerequisite for hypermagnesemia development (because of the high adaptability of Mg renal excretion), serum levels should be maintained within the normal values in stages 1–3 of CKD, secondary to the increase in fractional excretion of magnesium. As renal function declines beyond this threshold, the increased fraction excretion can no longer compensate, and hypermagnesemia is frequently noted in patients with a creatinine clearance <10 mL/min/1.73 m^2^ [22]. Although, theoretically, patients with advanced stages of CKD will rarely present reduced levels of serum Mg, a study performed by Oka et al. on 5126 pre-dialysis patients reported hypomagnesemia as the most common electrolyte abnormality, with similar prevalence across all CKD stages (approximately 15%) [65]. Furthermore, it is considered that the presence of hypomagnesemia in CKD patients may be explained by several pathophysiological mechanisms, such as tubular dysfunction or interstitial fibrosis, which contribute to an impaired tubular magnesium reabsorption [66]. In addition, proteinuria, a history of diabetes mellitus, and nonprescription of magnesium oxide were identified as risk factors. This suggests that some additional etiologies contribute to low serum Mg levels (such as extensive tubular injuries in advanced CKD stages and subsequent urinary Mg wasting) [65].

Another explanation for hypomagnesaemia in CKD is the correlation between Mg and vitamin D. Since intestinal absorption of Mg is influenced by vitamin D [67], and as a decrease in renal 1α-hydroxylase activity results in reduced 1,25-dihydroxyvitamin D (the major circulating active metabolite of vitamin D) serum levels in CKD [68,69], hypomagnesaemia can occur. Vitamin D loss due to heavy proteinuria has also been reported in early stages of CKD through loss of vitamin D-binding protein [70], emphasizing the possibility of also identifying hypomagnesemia in these particular conditions.

It was observed that 13.5–47.7% of type 2 diabetic patients can present hypomagnesemia [71], which could additionally be responsible for the progression and development of prediabetes [72]. Possible causes are the following [71]:

- Enhanced ultrafiltrable Mg because of glomerular hyperfiltration, hypoalbuminemia, or osmotic diuresis induced by hyperglycemia,
- Reduced Mg reabsorption in the thick ascending limb of the loop of Henle caused by insulin resistance or deficiency,
- Renal Mg waste at the proximal tubule and thick ascending limb of the loop of Henle caused by excessively vigorous volume expansion and glomerular hyperfiltration,
- Reduced oral intake and gastrointestinal absorption caused by diabetic autonomic neuropathies,
- Recurrent metabolic acidosis, hypophosphatemia, and hypokalemia linked with hypomagnesemia, etc.

Hypomagnesemia, as already mentioned, has also been associated with retinopathy, foot ulcers, poorer glycemic control [73], and a faster renal function deterioration rate [73,74] in patients with type 2 diabetes mellitus, and the risk of developing type 2 diabetes seems to be inversely proportional with magnesium intake (independent of other risk factors) in both men and women [75].

Signs and symptoms of magnesium depletion are represented by a wide array of biochemical abnormalities. Hypocalcemia is the most classical sign of severe hypomagnesemia. The mechanism includes a series of factors, such as low PTH secretion caused by a reduction in extracellular magnesium concentrations (without changes in calcium concentration), alongside an increase in PTH resistance. With a reported incidence between 40% and 60%, hypokalemia is also frequently associated with hypomagnesemia [76].

For a better understanding of the effects of hypomagnesemia on the progression of CKD, it should be highlighted that the disturbances in magnesium, along with calcium and phosphate homeostasis are incriminated by the CKD–MBD pathogenesis, determining the onset of uremic cardiomyopathy (Table 1), vascular calcifications, and mineral and bone abnormalities [77,78,79,80,81,82]. Furthermore, it is considered that the presence of hypomagnesemia in CKD patients (pre-dialysis and hemodialyzed population) can be highly predictive for an elevated risk of cardiovascular morbidity and mortality [77,78], as well as correlated with an increased risk of fractures [77,79,80,81].

Vascular calcifications in CKD develop secondary to the presence of hyperphosphatemia, hypomagnesemia, and decreased mineralization inhibitors (i.e., fetuin A, matrix gla protein, and osteoprotegerin) that contribute to the formation of calcioprotein particles and apatite deposits in the endothelium of vascular smooth muscle cells, generating inflammation, vasodilatation, oxidative stress, formation of matrix vesicles, and apoptosis [77]. Therefore, in vivo and in vitro studies showed the beneficial effect of magnesium supplementation [77,82]. In accordance with these data, a recent experimental study reported that the inhibition of MEMO 1 (mediator of cell motility 1—a redox protein that participates in the extracellular ligand-induced cell signaling) expression can stimulate the increase of serum magnesium level, which contributes to a decrease in serum calcification propensity, highlighting the beneficial effect of elevated serum levels against vascular calcification [83].

## 6. Hypomagnesemia in CKD Patients—Why Should We Care?

Now that we have established that hypomagnesemia is more frequent in CKD than we previously thought (starting with stage I of CKD), and considering that Mg deficiency has several negative effects (Table 2) [84], what are the implications for physicians and patients alike?

With a prevalence of almost 700 million cases in 2017, surpassing diseases such as diabetes, asthma, or depression, CKD is ranked as the 12th leading cause of death worldwide, surpassing HIV or tuberculosis. In that same year, CKD diagnosis resulted in 1.2 million deaths, a number expected to increase anywhere between 2.2 million and 4 million by 2040 [85,86].

It is a known fact that very high mortality and accelerated cardiovascular disease are associated with CKD, especially with end-stage renal disease (ESRD) [87]. In fact, a graded and independent correlation was reported between reduced estimated GFR and the risks of death, cardiovascular complications (i.e., coronary disease, heart failure, peripheral arterial disease, etc.), and hospitalization. These threats were noticed at an estimated GFR < 60 mL/min/1.73 m^2^ that increased significantly at an estimated GFR below 45 mL/min/1.73 m^2^ (with a staggering 343% risk increase at an eGFR <15 mL/min/1.73 m^2^) [88]. It is worth mentioning that microalbuminuria (defined as a 24 h urine albumin of 30–300 mg) represents another potential risk factor for different cardiac diseases and defects (i.e., left-ventricular hypertrophy and dysfunction, ischemic heart disease, etc.) [89], and it is also considered an increased overall risk factor for CVD [90]. Since CKD patients have a higher risk of undergoing a cardiovascular event than progressing to ESRD, the 2012 KDIGO guidelines recommend that all CKD patients be considered at increased risk for CVD [1].

As a common complication of CKD, vascular calcification, specifically arterial calcification, has been recognized for many years. The key element in vascular calcification pathogenesis appears to be the transformation of vascular smooth muscle cells (VSMC) into osteoblast-like cells, which may lead to smooth muscle calcification. Risk factors include hypertension, increasing age, hyperphosphatemia and calcium load, secondary hyperparathyroidism, vitamin D deficiency, increased FGF-23, and uremia. Arterial elasticity reduction, an increase in pulse wave velocity, left-ventricular hypertrophy, a decline in coronary artery perfusion, and myocardial ischemia are some of the hemodynamic implications of vascular calcification [91,92].

Calcification inhibitors include fetuin A (a hepatic plasma protein), matrix Gla protein (a vitamin K-dependent protein which seems to inhibit calcium crystal growth), osteoprotegerin (which inhibits osteoclast function), ectonucleotide pyrophosphatase phosphodiesterase, osteopontin, vitamin K, Klotho, and magnesium [93,94].

Magnesium seems to play a very important role when it comes to vascular calcification inhibition, as there is evidence that elevated serum Mg levels can decrease vascular calcification development [95,96]. An in vitro study, performed on bovine aortic vascular smooth cells, showed that Mg supplementation prevents hydroxyapatite crystal formation in the extracellular space (which seems to be essential for VSMC trans-differentiation) by preventing crystal nucleation [97]. In another in vitro study, on human aortic smooth muscle cells this time, Mg inhibited calcification through the inhibition of the phosphate-induced Wnt/b-catenin signaling pathway, and it could even reverse calcification lesions [98]. The expression of osteogenic transcription factors (BMP-2, RUNX2, Msh homeobox 2, and SRY-box 9), as well as bone proteins and genes associated with matrix mineralization (osteocalcin and alkaline phosphatase), is effectively counteracted by Mg supplementation. At the same time, Mg was observed to prevent the loss of inhibitors of calcification (BMP-7, matrix Gla protein, and osteopontin) that protect against osteogenic conversion. Furthermore, Mg has the capacity to block calcium channels in VSMCs (excessive intracellular calcium induces VSMC death and a subsequent release of apoptotic bodies, which promote matrix calcification) [82].

Different theories have been highlighted to explain the involvement of magnesium in preventing vascular calcification, by limiting the osteo-inductive expression [82].

- The inhibition of phosphate-induced Wnt/β-catenin signaling, a pathway that is induced by RUNX2 overexpression,
- The inhibition and improvement of deteriorated miRNAs (microRNA-30b, microRNA-133a, and microRNA-223) that are involved in the regulation of RUNX2, Smad1, and osterix (factors incriminated in the VSMC calcification);
- The activation of CaSR (calcium-sensing receptor), which regulates the ionic calcium influx in the VSMC; it is suggested that Mg could be considered as calcimimetic/gatekeeper that prevents the influx of ionic calcium.

In another study performed by Alesutan et al. [99], MgCl_2_ seemed to inhibit phosphate-induced osteogenic differentiation by activating the calcium-sensing receptor (which is less expressed in CKD patients). The calcium-sensing receptor, involved in regulating both parathyroid hormone and calcitonin secretion, is a G-protein-coupled cell surface receptor capable of sensing extracellular calcium ions, and a decrease in its expression in the vasculature has been directly linked with vascular calcification, as explained above [100]. A study performed on adenine-induced CKD rats showed that treatment with 750 mg/kg CaMg and 375 mg/kg CaMg reduced aortic calcification significantly [101], and magnesium treatment reduced vascular calcification by 43–52% in adenine-induced CKD rats treated with calcitriol [102].

In a prospective, observational study including 150 patients with CKD and diabetes, low serum Mg was associated with increased mitral valve calcification and increased carotid artery intima media thickness (IMT) [103], the latter being strongly associated with the risk of stroke and myocardial infarction [104,105]. Hypomagnesemia was also associated with increased carotid IMT in non-CKD patients [106]. In an interventional study featuring 72 patients on hemodialysis, Tzanakis et al. [107] showed that administration of 435 mg of calcium acetate containing 110 mg of elemental calcium combined with 235 mg of magnesium carbonate containing 60 mg of elemental magnesium for a 1 year period could slow and even regress arterial calcifications, and another interventional study featuring 51 patients on hemodialysis performed by Turgut et al. [108] showed that Mg supplementation improves carotid IMT. In addition, Mg supplementation can determine a better control of calcium and parathormone metabolism, which can contribute to the improvement of CKD–MBD [109].

Furthermore, a meta-analysis conducted by Liu et al. in 2021 that included 31 studies with a total of 205,436 participants diagnosed with CKD or ESRD showed a clear correlation between hypomagnesemia and the risk of cardiovascular diseases and mortality, especially in dialyzed patients [110,111]. Similar observations were noted by Wu et al.’s study, involving 169 HD patients, which reported a higher risk of mortality in hemodialyzed patients presenting lower serum Mg levels (below 1 mmol/L) [112].

A matter of debate remains the correlation of Mg serum levels and the risk of all-cause and cardiovascular mortality in CKD patients [110]. There are studies that reported the possibility of this type of association, but only one concluded that hypomagnesemia could be considered as an independent predictor for high risk of mortality in CKD patients [110,113]. Galán Carrillo et al.’s study, which included 746 patients with CKD stage 3 and 4, showed that hypermagnesemia can in fact be linked with an increased risk of cardiovascular diseases and all-cause mortality in CKD patients; therefore, Mg supplementation in this group of patients should be carefully and cautiously recommended [114]. In contrast to Galán Carrillo et al.’s observations, a recent in vivo study noted the beneficial effects of increased magnesium supplementation in the diet for preventing and decreasing the oxidative stress and proinflammatory response associated with CKD [115].

Furthermore, Xiong et al.’s meta-analysis, which included 20 studies with 200,934 subjects diagnosed with CKD and ESRD, noted that hypomagnesemia was in fact strongly correlated with cardiovascular and all-cause mortality in this group of patients [116]. Similar conclusions were reported by Jandaghi et al.’s study that showed the beneficial effects of increased levels of serum Mg in hemodialyzed patients [117].

Hypomagnesemia has also been linked to hypertension, as serum Mg increases prostacyclin and nitric oxide in the endothelium (both mediators of vasodilatation) [118]. There are in vitro and in vivo data showing the worsening of blood pressure values when low serum magnesium levels are presented; hypomagnesemia determined an increase of mean arterial pressure, total peripheral vascular resistance index, renal vascular resistance, and even myocardial metastatic tissue calcification (probably because of the reduced levels of Mg that cannot properly regulate the calcium passing through the cell channels, consequently inducing an elevation of intracellular calcium transfer) [119]. These findings highlight once more the need of a proper evaluation of serum magnesium in CKD patients, as hypertension is often associated with this population.

Magnesium seems to also be protective against the risk of high phosphate-derived CKD progression [120,121]. A retrospective study performed by Azem et al., which included 10,568 CKD patients (mean eGFR was 46.3 mL/min/1.73 m^2^), failed to demonstrate an association between serum Mg and CKD progression, but managed to link both hypomagnesemia and hypermagnesemia with an increase in all-cause mortality [122,123].

Considering the data previously mentioned, the fact that hypermagnesemia was often presented in critical ill patients [124], and the fact that clinically hypomagnesemia was correlated with a more accelerated disease progression [125], the adequate serum Mg level for reducing CKD progression is controversial as there is insufficient unified available information for this group of patients [126,127]. From our knowledge, the CRIC study, involving 5499 patients with CKD, is the only trial that recommended, on the basis of the performed analysis, the necessity of maintaining the serum Mg level between 1.9 and 2.1 mg/dL for reducing the risk of all-cause mortality in this population [128].

## 7. Conclusions

There is increased evidence of the importance of Mg in CKD progression. Although, theoretically, CKD patients with advanced stages of CKD should present exclusively hypermagnesemia, recent studies have indicated other pathophysiological mechanisms and risk factors leading to the onset of hypomagnesemia, which is linked with the risk of mortality at all stages of CKD. Additionally, it may contribute to vascular calcification development, elevated blood pressure, risk of myocardial infarction, stroke, etc. Nevertheless, there are studies that failed to notice a strong association between hypomagnesemia and CKD progression, and others that linked hypermagnesemia to an increased risk of cardiovascular diseases and all-cause mortality in the CKD population. A new study highlighted the idea that, in patients with CKD, a level of serum Mg between 1.9 and 2.1 mg/dL could reduce the risk of all-cause mortality. Therefore, further studies are necessary to validate and unify these findings that could be useful in developing a better, more adequate, and personalized management of CKD patients.

## Figures and Tables

**Table 1:** Incriminated factors in the onset of uremic cardiomyopathy [82].

Traditional Factors
Hypertension
Diabetes mellitus
Dyslipidemia
Smoking
**Specific Factors**
Secondary hyperparathyroidism
Anemia
Malnutrition
Anemia
**New Risk Factors**
Hyperphosphatemia
Hypomagnesemia
Increased FGF-23 level
Decreased α-Klotho

FGF-23: fibroblast growth factor 23. Modified after Ferrèet al. Calcium, Phosphate, and Magnesium Metabolism in Chronic Kidney Disease. *Chronic Renal Disease*. Academic Press, 2020, pp. 661–679.

**Table 2:** The consequences of Mg deficiency [84].

Pathophysiological Effects
Accelerated atherosclerosis
Proinflammatory effect
Increased thromboxane synthesis
Increased synthesis of cytokines, nitric oxide
Aldosterone overproduction
Sympathetic nervous system overactivity
**Diseases**
Hypertension
Cardiovascular diseases
Renal impairment

Modified after Rodelo-Haad et al. The Role of Disturbed Mg Homeostasis in Chronic Kidney Disease Comorbidities. *Front. Cell Dev. Biol.* **2020**, *8*, 543099.

## Data Availability

Not applicable.
